# *Withania somnifera* Extract Enhances Energy Expenditure via Improving Mitochondrial Function in Adipose Tissue and Skeletal Muscle

**DOI:** 10.3390/nu12020431

**Published:** 2020-02-07

**Authors:** Da-Hye Lee, Jiyun Ahn, Young-Jin Jang, Hyo-Deok Seo, Tae-Youl Ha, Min Jung Kim, Yang Hoon Huh, Chang Hwa Jung

**Affiliations:** 1 Division of Food Functionality Research, Korea Food Research Institute, Wanju-gun, Jeonbuk 55365, Korea; leedahye0826@gmail.com (D.-H.L.); jyan@kfri.re.kr (J.A.); jyj616@kfri.re.kr (Y.-J.J.); hyo-deok.seo@kfri.re.kr (H.-D.S.); tyhap@kfri.re.kr (T.-Y.H.); kmj@kfri.re.kr (M.J.K.); 2 Department of Food Biotechnology, University of Science and Technology, Daejeon 34113, Korea; 3 Center for Electron Microscopy Research, Korea Basic Science Institute, Cheongju 28119, Korea; hyh1127@kbsi.re.kr

**Keywords:** *Withania somnifera*, energy expenditure, mitochondrial activity, browning, withaferin A, anti-obesity

## Abstract

*Withania somnifera* (WS), commonly known as ashwagandha, possesses diverse biological functions. WS root has mainly been used as an herbal medicine to treat anxiety and was recently reported to have an anti-obesity effect, however, the mechanisms underlying its action remain to be explored. We hypothesized that WS exerts its anti-obesity effect by enhancing energy expenditure through improving the mitochondrial function of brown/beige adipocytes and skeletal muscle. Male C57BL/6J mice were fed a high-fat diet (HFD) containing 0.25% or 0.5% WS 70% ethanol extract (WSE) for 10 weeks. WSE (0.5%) supplementation significantly suppressed the increases in body weight and serum lipids, and lipid accumulation in the liver and adipose tissue induced by HFD. WSE supplementation increased oxygen consumption and enhanced mitochondrial activity in brown fat and skeletal muscle in the HFD-fed mice. In addition, it promoted browning of subcutaneous fat by increasing mitochondrial uncoupling protein 1 (UCP1) expression. Withaferin A (WFA), a major compound of WS, enhanced the differentiation of pre-adipocytes into beige adipocytes and oxygen consumption in C2C12 murine myoblasts. These results suggest that WSE ameliorates diet-induced obesity by enhancing energy expenditure via promoting mitochondrial function in adipose tissue and skeletal muscle, and WFA is a key regulator in this function.

## 1. Introduction

Obesity is caused by an imbalance between energy intake and energy expenditure. When the energy expenditure exceeds the intake, this leads to weight loss [1]. Energy expenditure comprises the basal metabolic rate, physical activity (exercise-induced), and adaptive thermogenesis (shivering, non-shivering, and diet-induced) [2]. Recent studies have suggested that adaptive thermogenesis is a new therapeutic approach to treating obesity [3]. Adaptive thermogenesis occurs mainly in brown adipose tissue (BAT) and skeletal muscle [4]. The various phytochemicals in medicinal plants have been reported to ameliorate obesity through promoting mitochondrial biogenesis and thermogenesis in adipose tissue [5,6].

BAT is composed of multilocular adipocytes that release energy through non-shivering thermogenesis facilitated by the mitochondrial uncoupling protein 1 (UCP1) [7] and is considered a potential target for the treatment of obesity [8]. White adipose tissue (WAT), especially subcutaneous WAT, can transform into brown-like adipocytes (beige adipocytes) in response to a thermogenic stimulant, such as cold exposure, or β3-adrenergic or peroxisome proliferator-activated receptor gamma (PPAR-γ) agonists [9,10]. Beige adipocytes contain multilocular lipid droplets and are abundant in mitochondria. These adipocytes also have increased expression of BAT-specific genes, such as *UCP1*, cell death activator CIDE-A (*Cidea*), and peroxisome proliferator-activated receptor gamma coactivator 1-alpha (*PGC1α*), in response to thermogenic activation [9]. Skeletal muscle is also involved in adaptive thermogenesis as it induces shivering thermogenesis via mitochondrial uncoupling in cold conditions [11,12]. 

Mitochondria contain respiratory complexes (I–IV) in the inner membrane, which function as the sites of oxidative phosphorylation by pumping protons from the matrix to the intermembrane space [13]. Reactive oxygen species (ROS) are generated as a by-product of mitochondrial respiration [14], and oxidative damage to mitochondria induces lipid accumulation by reducing electron transport chain function and fatty acid oxidation [15]. When nutrients are consumed in excess, saturated free fatty acids accumulate, leading to mitochondrial dysfunction and insulin resistance [16]. Mitochondrial dysfunction is correlated with metabolic disorders such as obesity and type 2 diabetes. Improvement of the mitochondrial function in metabolic tissues is a known therapeutic approach for the treatment of metabolic disorders [17]. 

*Withania somnifera* (WS), also known as ashwagandha or Indian ginseng, has been traditionally used in indigenous medicine to improve chronic fatigue and promote youthful vigor [18]. WS possesses anticancer, anti-inflammatory, antioxidative, and antistress properties [19,20] and contains diverse phytochemicals such as alkaloids, steroidal lactones, and steroids [18]. Although previous studies have demonstrated that WS suppresses body weight gain induced by chronic stress [21], the underlying mechanism has yet to be explored. WS has been reported to enhance muscle activity by increasing muscle strength and mass [22,23]. Improving the activity of skeletal muscle implies the possibility of increasing energy expenditure. In addition, plant alkaloids contained in WS have been reported that promote browning of adipose tissue [5,24,25]. In this regard, WS appears to be a therapeutic candidate to improve energy expenditure by increasing adaptive thermogenesis. 

In the current study, we hypothesized that WS prevents obesity by increasing energy expenditure through enhancing activity of mitochondria in tissues with high energy metabolism. We here aimed to evaluate the energy expenditure-enhancing effect of WSE (WS 70% ethanol extract) in diet-induced obese mice and elucidate the underlying mechanism with determination of the mitochondrial activity in skeletal muscle and adipose tissue.

## 2. Materials and Methods

### 2.1. WS Extract (WSE) Preparation

WS root powder (Herbs India, Coimbatore, India) was extracted with 70% ethanol at 80 °C for 2 h. The extract was filtered through Whatman No. 2 filter paper, concentrated using a vacuum evaporator, and lyophilized using a freeze dryer.

### 2.2. Materials

Dulbecco’s modified Eagle’s medium, calf serum, fetal bovine serum (FBS), penicillin–streptomycin, and phosphate-buffered saline were obtained from Gibco BRL (Grand Island, NY, USA). Antibodies against-β-actin (sc-47778), type 2 deiodinase (DIO2; sc-98716), and uncoupling protein 2 (UCP2; sc-6526), and secondary antibodies were purchased from Santa Cruz Biotechnology (Santa Cruz, CA, USA). Antibody against voltage-dependent anion channel (VDAC; 4661s) was purchased from Cell Signaling Technology (Danvers, MA, USA). Antibodies against UCP1 (ab23841) and total oxidative phosphorylation (OXPHOS) complex (ab110413) were purchased from Abcam (Cambridge, MA, USA). Antibody against total myosin heavy chain was purchased from Developmental Studies Hybridoma Bank (Iowa city, IA, USA). 3-Isobutyl-1-methylxanthine (IBMX, l7018), withaferin A (WFA; W4394), withanolide A (WNA; W2145), and dexamethasone (D4902) were purchased from Sigma-Aldrich Chemical Co. (St. Louis, MO, USA). Radioimmunoprecipitation assay buffer (89900) and protease- and phosphatase-inhibitor cocktails (78440) were purchased from Thermo Scientific-Pierce (Rockford, IL, USA).

### 2.3. Animals

Four-week-old male C57BL/6J mice were purchased from Japan SLC Inc. (Hamamatsu, Japan). Animal studies were conducted in accordance with institutional and national guidelines, and all experimental procedures were approved by the Korea Food Research Institute Animal Care and Use Committee (KFRI-IACUC, KFRI-M-16054). Mice were divided into four groups: a normal group (*n* = 10) fed American Institute of Nutrition Rodent Diet AIN-76, a group fed a high-fat diet (HFD group, *n* = 10), and two groups fed HFD with either 0.25% or 0.5% WSE (HFD + WSE 0.25% or 0.5% groups, each *n* = 10). The experimental diets were based on the AIN-76 diet and contained 45% fat and 0.5% cholesterol (*w*/*w*) (Supplementary Table S1). After consuming the respective diets for 10 weeks, all mice were anesthetized with 2% isoflurane, sacrificed, and the liver, muscle, and adipose tissues were harvested and weighed. Some tissues were fixed in 4% formaldehyde, and the rest were stored at −80 °C for further analysis. Blood was collected from the abdominal aorta and centrifuged at 3000 rpm for 20 min at 4 °C to separate serum.

### 2.4. Energy Expenditure Measurement

Energy expenditure was measured at 10 weeks using an OxyletPro^TM^ system (Panlab Harvard Apparatus, Barcelona, Spain). Mice were placed and acclimated in an individual metabolic chamber for 24 h, and then we measured oxygen consumption (VO_2_) and carbon dioxide production (VCO_2_) over 24 h using Oxylet LE 405 gas analyzer (Panlab Harvard Apparatus, Barcelona, Spain). The animals were maintained at 22 °C in a 12 h light/dark cycle with free access to food and water. At each measurement, OxyletPro Metabolism V3.0 software (Panlab Harvard Apparatus, Barcelona, Spain) automatically calculated the respiratory quotient (RQ) as the VO_2_/VCO_2_ ratio and energy expenditure (EE) as VO_2_ × 1.44 × [3.815 + (1.232 × RQ)] (kcal/day/kg^0.75^) according to the Weir formula [26].

### 2.5. OGTT and IPITT

Oral glucose tolerance test (OGTT) and intraperitoneal insulin tolerance test (IPITT) were performed in response to oral glucose (2 g/kg, G5146, Sigma-Aldrich) and insulin injection (0.75 unit/kg, 11376497001, Roche, Australia) after fasting for 4 h, respectively. Blood samples were collected from the tail vein at 0, 15, 30, 60, and 120 minutes. Blood glucose concentrations were determined using an Accu-Chek glucometer (Roche, Mannheim, Germany). 

### 2.6. Quantitative Reverse-Transcription Polymerase Chain Reaction (RT-qPCR)

Total RNA was extracted from tissues and cells using RNeasy mini kit (Qiagen, Valencia, CA, USA). cDNA was synthesized using the ReverTra Ace® qPCR RT Kit (Toyobo, Osaka, Japan). qPCRs were run in an ViiA^TM^ 7 Real-time PCR system (Applied Biosystems, Foster, CA, USA) using SYBR Green Real-time PCR Master Mix (Toyobo, Osaka, Japan) and the following thermal cycles: 95 °C for 1 min, 40 cycles of 95 °C for 15 s, 60 °C for 15 s, and 75 °C for 45 s. The gene-specific primers are listed in Supplementary Table S2.

### 2.7. Immunoblotting

Cells and tissues were lysed in RIPA buffer supplemented with protease- and phosphatase-inhibitor cocktails. Proteins were then separated by sodium dodecyl sulfate-polyacrylamide gel electrophoresis and transferred to polyvinylidene fluoride membranes. The membranes were blocked and then incubated with primary and secondary antibodies. The protein levels were visualized using a chemiluminescence reagent.

### 2.8. Immunofluorescence Staining of UCP1

Beige adipocytes were fixed with 4% formaldehyde, permeabilized with 0.5% saponin, blocked with 1% bovine serum albumin, and incubated with UCP1 antibody. The antibody was visualized using a fluorescein isothiocyanate secondary antibody (Alexa Fluor 488), and the nuclei were stained with 4′,6-diamidino-2-phenylindole. The stained cells were visualized with a Nikon Eclipse Ti confocal laser scanning microscope (Nikon, Tokyo, Japan).

### 2.9. Immunohistochemistry of UCP1

Tissues were fixed in 10% formalin, and the endogenous peroxidase activity was inhibited by incubating with 0.3% H_2_O_2_ in PBS. The fixed tissues were blocked by 1% horse serum and incubated with UCP1 antibody. UCP1 expression was visualized using a diaminobenzidine solution, and the slides were mounted and examined under an Olympus TH4-200 microscope (Tokyo, Japan).

### 2.10. Histologic Analysis 

Tissues were fixed in 10% formalin, embedded in paraffin, cut into 5 µm sections, and stained with H&E; their morphologies were evaluated using an Olympus TH4-200 microscope (Tokyo, Japan). 

### 2.11. TEM

Skeletal muscle was immediately fixed in 2% glutaraldehyde and 2% paraformaldehyde in 0.1 M PBS (pH 7.4). After washing with PBS, the tissues were post-fixed with 1% osmium tetroxide and washed again with PBS. The tissues were then embedded in pure Epon 812 mixture after dehydration in ethanol series and infiltration in a propylene oxide:epon mixture series. Ultrathin sections (~70 nm) were obtained with a model MT-X ultramicrotome (RMC, Tucson, AZ, USA) and then stained with 2% uranyl acetate and lead citrate. The sections were visualized by Cryo-TEM (JEM-1400 Plus, 120 kV) (Jeol Ltd., Tokyo, Japan).

### 2.12. Measurement of Complex III and IV Activity 

Mitochondrial fraction was isolated from skeletal muscle using the mitochondria isolation kit (ab110168; Abcam), and the activity of complex III and complex IV from isolated mitochondria was measured using a microplate assay kit (ab109905 and ab109911 respectively; Abcam) according to manufacturer’s instruction.

### 2.13. LC-MS/MS Analysis

The analyses were performed using an Acquity UPLC system (Waters, Miliford, MA, USA) with Acquity UPLC BEH C18 column (2.1 mm × 100 mm, 1.7 µm). The mobile phase included 0.1% formic acid aqueous solution (Solvent A) and 0.1% formic acid in acetonitrile (Solvent B), and a gradient elution program was performed: 0–5.5 min, 50%–20% solvent A; 5.5–7 min, 20%–80% solvent A; 7–8 min, 2%–98% solvent A; 8–10 min, 50% solvent A, and 10–11 min, isocratic 50% solvent A. The flow rate was set at 0.5 mL/min, and column temperature was kept at 40 °C. The autosampler was conditioned at 4 °C, and the injection volume was 5 μL. Mass spectrometric analyses were operated using a Waters Xevo TQ triple-quadrupole mass spectrometer equipped with electrospray ionization (ESI) mode.

### 2.14. Cell Culture and Treatment

3T3-L1 mouse fibroblasts (CL-173) and C2C12 cells (CRL-1772) were purchased from the American Type Culture Collection and were cultured in DMEM containing 10% calf serum or 10% FBS in a 5% CO_2_ incubator maintained at 37 °C. The cells were treated with a different concentration (0.125−0.5 µM) of WFA and WNA, respectively. 

### 2.15. Beige Adipocyte Differentiation of 3T3-L1 Cells

3T3-L1 cells were cultured to 100% confluence. Confluent 3T3-L1 cells (day 0) were incubated in DMEM containing 0.25 μM dexamethasone, 0.5 μM IBMX, 10 µg/mL insulin, and 50 nM triiodothyronine (T_3_), and 10% FBS for 2 days. The cells were then incubated in 10% FBS-DMEM containing 0.5 μM IBMX, 1 µg/mL insulin, 50 nM T_3_, and 1 μM rosiglitazone for 6 days.

### 2.16. Determination of Oxygen Consumption Rate (OCR) in C2C12 Cells

Oxygen consumption was analyzed using an XF24 extracellular flux analyzer (Seahorse Bioscience, North Billerica, MA, USA). Briefly, C2C12 cells were seeded at 2 × 10^5^/mL in a Seahorse 24-well plate and treated with WFA or WNA for 24 h. The culture medium was changed and incubated with XF assay medium (102353-100, Agilent Technologies, Santa Clara, CA, USA) supplemented with 0.25 mM glucose, 1 mM pyruvate, and 4 mM l-glutamine at 37 °C for 1 h in a non-CO_2_ incubator. The OCR was analyzed with a XF cell mito stress kit (103015-100, Agilent Technologies, Santa Clara, CA, USA) by sequential injection of 1 μM oligomycin A, 2 μM carbonyl cyanide-4-(trifluoromethoxy) phenylhydrazone (FCCP), and 1 μM rotenone. The basal respiration rate, ATP production, maximal respiration, and spare capacity was calculated by Seahorse XF software version 1.8.1.

### 2.17. Statistical Analysis

Data are expressed as the mean ± standard deviation (SD) or standard error of the mean (SEM). Means were compared by one-way analysis of variance (ANOVA) followed by Tukey’s post hoc test, using Prism7 software (GraphPad Software, San Diego, CA, USA). A *p*-value < 0.05 was considered significant.

## 3. Results

### 3.1. WSE Prevents HFD-Induced Obesity in Mice by Enhancing Energy Expenditure

**Figure 1:**
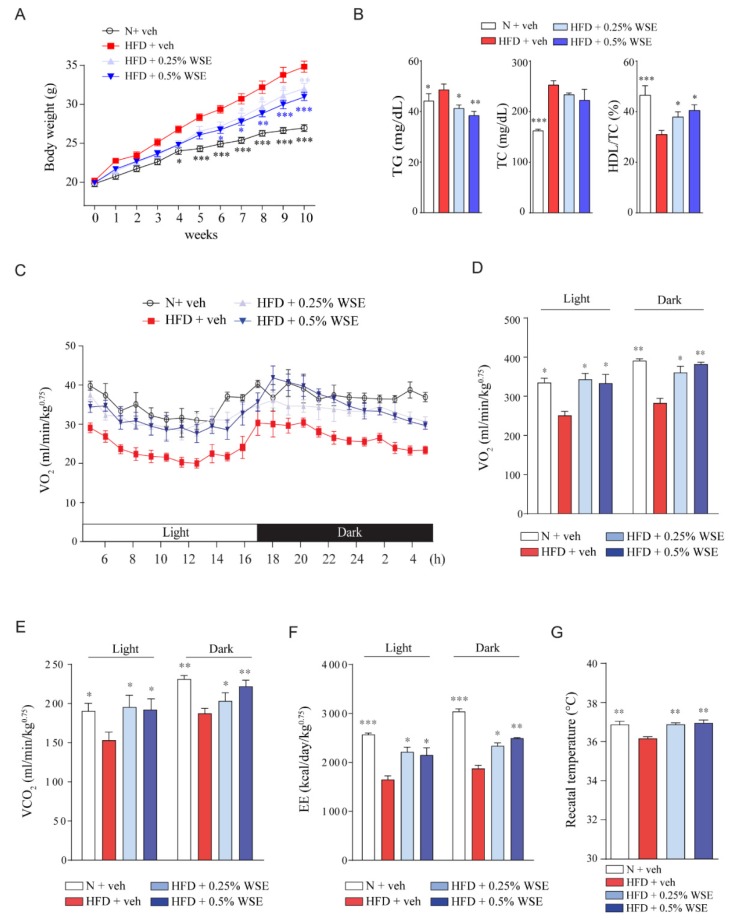
**WSE prevents obesity by enhancing the oxygen consumption rate (OCR) in mice fed an HFD (high-fat diet).** (**A**) Effect of WSE on mouse body weight during the 10 weeks experimental period. (**B**) Serum lipid levels. (**C**) VO_2_ levels throughout the light/dark cycle were analyzed by indirect calorimetry. The levels were normalized to body weight. (**D**) Area under the curve (AUC) of VO_2_. AUC was calculated using Prism software (ΔX*(Y1 + Y2)/2, X: Value of *X* axis, Y: Value of *Y* axis). (**E**) AUC of VCO_2_. (**F**) Energy expenditure was calculated based on the VO_2_ and VCO_2_ levels. (**G**) Rectal temperature was measured at room temperature. Data represent the mean ± SEM (*n* = 5). Difference between groups was evaluated by Tukey’s multiple comparison test. * *p* < 0.05; ** *p* < 0.01; *** *p* < 0.001 compared with the HFD group. N: Normal control diet.

We investigated whether WSE supplementation would exhibit an anti-obesity effect in HFD-fed mice. HFD effectively induced a body weight gain at 10 weeks, and the two HFD + WSE groups (0.25% or 0.5% WSE) had a significantly reduced body weight gain when compared with the HFD group (Figure 1A). Food intake per day was not different in all groups (Supplementary Figure S1A). WSE supplementation significantly suppressed the increase in serum triglyceride induced by HFD, and the serum high-density lipoprotein/total cholesterol ratio was significantly increased by this supplementation (Figure 1B). Both WSE + HFD groups showed decreased liver and white adipose tissue (WAT) weights, but increased muscle per body weight when compared with the HFD group (Supplementary Figure S1B). When we measured adipocyte size using H&E staining, we found that WSE supplementation significantly reduced adipocyte size in epididymal WAT when compared with the size observed in the HFD group (Supplementary Figure S1C,D). WSE supplementation also reduced hepatic lipid accumulation (Supplementary Figure S1E,F) and the expression of lipid metabolism-related genes, such as cluster of differentiation 36 (*CD36*), stearoyl-CoA desaturase 1 (*SCD1*), and sterol regulatory element-binding protein 1c (*SREBP1c*) at 10 weeks (Supplementary Figure S1G).

We evaluated the effect of WSE on insulin resistance in HFD-fed mice using an oral glucose tolerance test (OGTT) and intraperitoneal insulin tolerance test (IPITT). The 0.5% WSE supplementation, but not the 0.25% WSE, showed a significant reduction in the glucose level in OGTT when compared with the HFD group (Supplementary Figure S2A,B). In the IPITT, the 0.25% and 0.5% WSE groups showed a significant reduction in the glucose level when compared with the HFD group (Supplementary Figure S2C,D). Taken together, these finding showed that WSE exhibited an anti-obesity effect and improved insulin resistance in the HFD-fed mice.

WS reportedly enhances muscle activity by increasing muscle mass and strength [22,23]. Therefore, we hypothesized that WSE enhances energy expenditure by increasing muscle activity. Thus, we measured oxygen consumption (VO_2_) and carbon dioxide production (VCO_2_) using the OxyletPro system to calculate the energy expenditure. Both HFD + WSE groups showed increased VO_2_ and VCO_2_ levels and energy expenditure when compared with the HFD group (Figure 1C–F). WSE supplementation significantly reversed the rectal temperature decrease caused by HFD at 24 °C (Figure 1G). These results indicate that WSE may reduce the body weight gain induced by HFD by increasing oxygen consumption and thermogenesis.

### 3.2. WSE Enhances BAT Activity and Browning of WAT in HFD-Fed Mice

**Figure 2:**
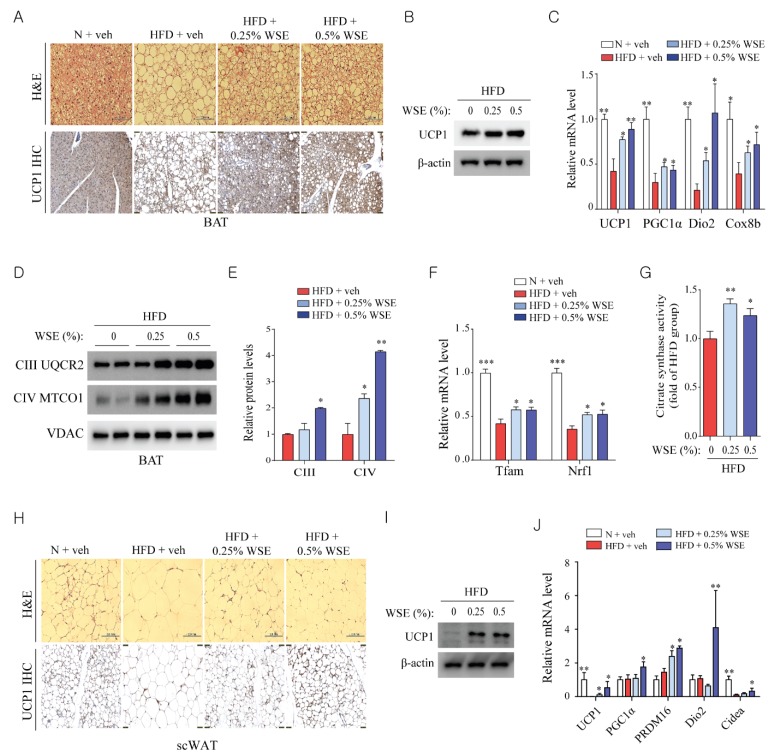
WSE enhances the mitochondrial function of brown adipose tissue (BAT) and the browning of subcutaneous adipose tissue (scWAT) in HFD-fed mice. (**A**) H&E staining and immunohistochemistry for the quantification of UCP1 (uncoupling protein 1) in BAT. (**B**) Protein expression of UCP1 in BAT. (**C**) Relative mRNA expression of BAT-specific genes (*n* = 5). (**D**) Expression of proteins involved in mitochondrial complexes III and IV in BAT. The subunits of mitochondrial complexes were detected using the total OXPHOS (oxidative phosphorylation) complex antibody cocktail. (**E**) Densitometry-based quantification data in (D), normalized to the level of VDAC (voltage-dependent anion channel). (**F**) Relative mRNA levels of the mitochondrial biogenesis-related genes, Tfam and Nrf1 (*n* = 5). (**G**) Citrate synthase activity in BAT (*n* = 5). (**H**) H&E staining and immunohistochemistry for quantification of UCP1 expression in scWAT. (**I**) Protein levels of UCP1 in scWAT. (**J**) mRNA expression of BAT-specific genes in scWAT (*n* = 5). Data represent the mean ± SEM. Difference between groups was evaluated by Tukey’s multiple comparison test. * *p* < 0.05; ** *p* < 0.01; *** *p* < 0.001 compared with the HFD group.

BAT is rich in mitochondria, which are essential for non-shivering thermogenesis [9]. H&E staining of BAT showed that WSE supplementation resulted in a reduction in lipid accumulation induced by HFD (Figure 2A). Immunohistochemical and immunoblot analyses revealed that UCP1 expression was increased by WSE supplementation when compared with the level in mice fed the HFD (Figure 2A,B). The HFD decreased the expression of BAT-enriched genes, including *UCP1*, *PGC1α*, *Dio2*, and cytochrome c oxidase subunit 8b (*Cox8b*) in BAT; however, this effect was reversed by WSE supplementation (Figure 2C). WSE supplementation also increased protein expression related to mitochondrial complexes III and IV in BAT, when compared with the levels of these proteins in the HFD group (Figure 2D,E), and mRNA expression of mitochondrial transcription factor A (*Tfam*) and nuclear respiratory factor 1 (*Nrf1*), which are related to mitochondrial biogenesis (Figure 2F). Both HFD + WSE groups showed increased citrate synthase activity in BAT when compared with the HFD group (Figure 2G). These results suggested that WSE enhances mitochondrial activity in BAT.

Subcutaneous WAT (scWAT) can be differentiated into beige adipocytes, which are brown fat-like cells that are induced upon exposure to prolonged cold conditions or β-adrenergic receptor activation. Beige adipocytes are rich in mitochondria and express BAT-specific proteins, such as UCP1 [27]. We investigated whether scWAT could differentiate into beige adipocytes by WSE supplementation in HFD-fed mice. WSE supplementation decreased the adipocyte size and increased UCP1 expression when compared with the levels observed in the HFD group (Figure 2H,I). WSE supplementation also significantly increased the mRNA levels of BAT-specific genes, including *UCP1*, *PGC1α*, PR domain containing 16 (*Prdm16*), *Dio2*, and *Cidea*, when compared with those in the HFD group (Figure 2J). These results indicated that WSE enhances the differentiation of scWAT to BAT-like adipocytes. 

### 3.3. WSE Improves Mitochondrial Function in Skeletal Muscle

**Figure 3:**
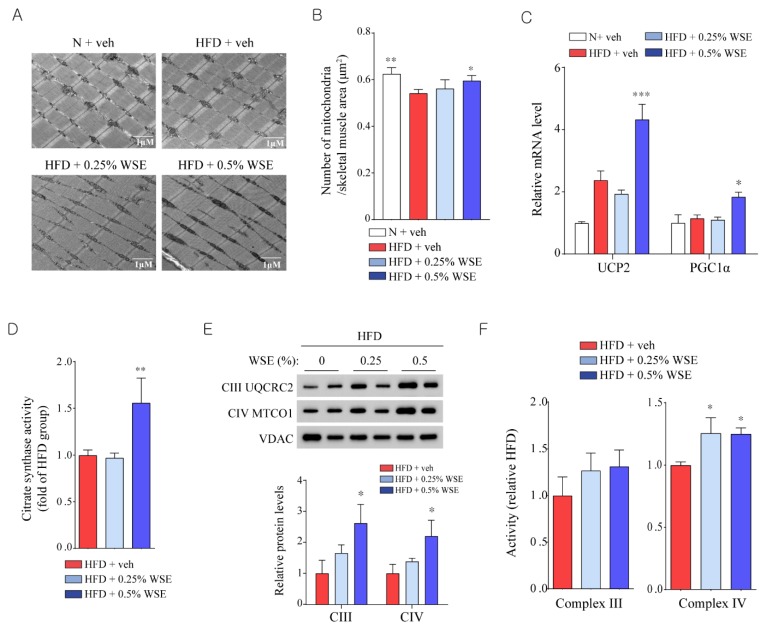
**WSE improves mitochondrial function in skeletal muscle.** (**A**) Representative transmission electron microscopic images of the skeletal muscle. (**B**) Number of mitochondria per skeletal muscle area. (**C**) Relative mRNA levels of mitochondrial-related genes in skeletal muscle. (**D**) Citrate synthase activity in skeletal muscle. (**E**) Expression of protein involved in mitochondrial complexes III and IV in skeletal muscle, and densitometry-based quantification of the data, normalized to the level of VDAC (*n* = 3). (**F**) Activity of mitochondrial complexes III and IV in skeletal muscle. Data represent the mean ± SEM (*n* = 5). Difference between groups was evaluated by Tukey’s multiple comparison test. * *p* < 0.05; ** *p* < 0.01; *** *p* < 0.001 compared with the HFD group.

We counted mitochondria in skeletal muscle using electron microscopy. Although both the 0.25% and 0.5% WSE groups had an increased number of mitochondria when compared with the HFD group (Figure 3A,B), only the 0.5% HFD + WSE group showed a significant increase in the mRNA levels of mitochondria-related genes, including *UCP2* and *PGC1α* (Figure 3C). We measured citrate synthase activity as a marker for aerobic capacity and mitochondrial density in skeletal muscle. Of the two groups of WSE, only the 0.5% WSE group showed a significant increase in mitochondrial citrate synthase activity when compared with the HFD group (Figure 3D). Mitochondria have respiratory chain complexes consisting of complexes I–IV [13]. Complex III (cytochrome bc 1) and complex IV (cytochrome c oxidase) transport protons to the outside of the mitochondrial inner membrane, leading to the production of ATP through an electrochemical gradient [13]. WSE supplementation tended to increase the expression of proteins involved in mitochondrial complexes III and IV (Figure 3E) in the mitochondrial fraction of skeletal muscle. The activity of complex IV, but not complex III, was significantly increased in the two WSE-supplemented groups (Figure 3F). These results suggested that 0.5% WSE supplementation improves mitochondrial activity in the skeletal muscle of HFD-fed mice.

### 3.4. WFA Enhances the Differentiation of 3T3-L1 Cells into Beige Adipocytes and Mitochondrial Function in C2C12 Cells

**Figure 4:**
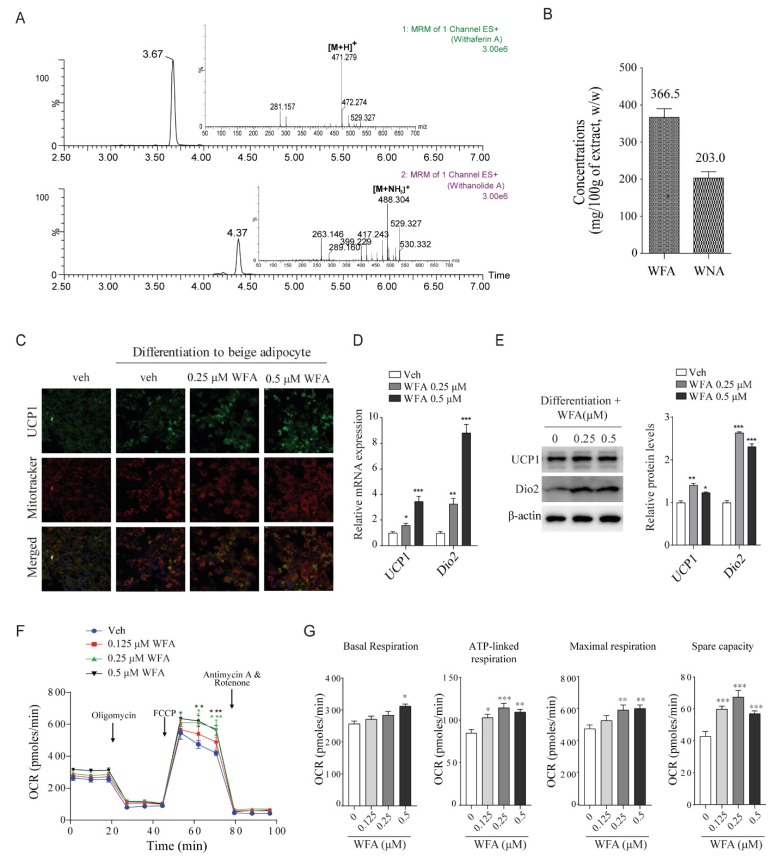
**Withaferin A (WFA) in WS enhances the differentiation into beige adipocytes and increases OCR.** (**A**) LC-MS/MS analysis of WSE. (**B**) Contents of WFA and withanolide A (WNA) in 100 g of WSE. (**C**) UCP1 expression in differentiated 3T3-L1 beige adipocytes. (**D**) Expression of brown adipocyte-specific mRNA in differentiated 3T3-L1 beige adipocytes. (**E**) WFA increases protein expressions of UCP1 and Dio2 in beige adipocytes (*n* = 3). (**F**) WFA increases the OCR in C2C12 cells. C2C12 cells were treated with the indicated concentrations of WFA for 24 h. (**G**) Quantification of basal respiration, ATP production, maximal respiration, and spare capacity. Data represent mean ± SD of three independent experiments. Difference between groups was evaluated by Tukey’s multiple comparison test. * *p* < 0.05; ** *p* < 0.01, *** *p* < 0.001 compared with the vehicle group.

We performed LC-MS/MS analysis to determine the compound(s) in WSE responsible for its energy expenditure-enhancing effect. LC-MS/MS analysis revealed that WFA and WNA are both present in WSE (Figure 4A). Per 100 mg of WSE, we detected approximately 366 µg of WFA and 203 µg of WNA (Figure 4B). We investigated whether WFA and WNA promote differentiation into beige adipocytes, using the 3T3-L1 cells. Treatment with WFA, but not WNA (data not shown), increased the expression of UCP1, which co-localized with mitochondria during the differentiation into beige adipocytes (Figure 4C). WFA increased the mRNA and protein expression of browning-related genes, including *UCP1* and *Dio2*, in the 3T3-L1 cells (Figure 4D,E). These results showed that WFA induces the differentiation of pre-adipocytes into beige adipocytes.

Finally, we measured whether WFA enhances the OCR in C2C12 mouse myoblasts. WFA promoted the OCR in a dose-dependent manner (Figure 4F) and elevated, albeit insignificantly, the OCR level of basal respiration (Figure 4G). WFA also increased the ATP turnover capacity after oligomycin treatment, which inhibits ATP synthesis by blocking complex V. WFA increased the maximal respiration by FCCP treatment and spare capacity. WNA also increased OCR in C2C12 cells (Supplementary Figure S3A–E) and increased the protein expression of myosin heavy chain and UCP2 in the C2C12 cells (Supplementary Figure S3F). These results indicated that WNA as well as WFA may regulate energy expenditure in vivo. 

## 4. Discussion

Increasing the metabolic rate through inducing adaptive thermogenesis is a promising alternative strategy for the treatment of obesity [9]. Phytochemicals, including resveratrol, berberine, and caffeine, have been demonstrated to increase thermogenesis through stimulating mitochondrial biogenesis and expression of UCP1 in adipose tissue [28,29,30,31]. The development of dietary phytochemicals responsible for inducing adaptive thermogenesis has attracted considerable attention. WS reportedly increases the strength and endurance of muscle, where adaptive thermogenesis mainly occurs, and ameliorates the chronic stress-related obesity [21,22,23]; however, its potential anti-obesity effect had not been fully explored. Therefore, we investigated whether WSE would prevent HFD-induced obesity in mice. This study is the first to demonstrate the effect of enhancing energy expenditure of WSE. Here, we have identified that WSE enhances mitochondrial function in BAT and skeletal muscle, and promotes browning of scWAT. In addition, it was also found that WFA in WSE promotes differentiation into beige adipocytes of 3T3-L1 pre-adipocyte and increases OCR in myocytes. 

Our results first showed that WSE suppressed the body weight gain and lipid accumulation in the liver and epididymal WAT (eWAT) induced by HFD, suggesting that it prevents obesity. There was no difference in food intake between the groups and no adverse effects. Also, treatment of WSE in 3T3-L1 cells did not affect cell viability up to 120 μg/mL (data not shown), indicating that these effects have not resulted from the toxicity of the WSE. We hypothesized that WSE increases the energy expenditure by enhancing muscle activity, or via the activation of another tissue function that is related to energy metabolism. To identify potential energy expenditure-enhancing effects, we indirectly measured OCR using a calorimeter. It has been reported that HFD decreases energy expenditure by suppressing oxygen consumption [32]. We found that WSE increased energy expenditure by increasing oxygen consumption and thermogenesis in HFD-fed mice. The increased rectal temperature by WSE supplementation indicates an enhanced dissipation of energy through heat generation [33]. 

The mitochondrion is a central organelle in energy metabolism, as it is the site of ATP production and is involved in the regulation of energy expenditure [34]. Reduced mitochondrial oxidative capacity leads to metabolic inflexibility [35]. WSE supplementation enhanced the activity of citrate synthase, mitochondrial enzyme commonly used as marker for mitochondrial content [36], which catalyzes citrate formation by adding oxaloacetate to acetyl-CoA in the tricarboxylic acid cycle in skeletal muscle and BAT. WSE increased mitochondrial respiratory complex subunit expression, and complex activity in the skeletal muscle of the HFD + WSE groups. In addition, WSE supplementation increased mitochondrial biogenesis-related gene expression in skeletal muscle and BAT. These results suggest that WSE may increase the metabolic rate by improving mitochondrial activity. Irisin, a hormone secreted mainly in skeletal muscle, has been described to promotes thermogenesis and browning of WAT via increasing the expression of UCP1 in adipose tissue [37]. Since skeletal muscle and adipose tissue have functionally interacted via secreted proteins, thus WSE may contribute to improving overall mitochondrial function [38]. 

Enhancement of energy expenditure is an emerging potential therapeutic target for weight loss [5]. BAT, one of the high-energy-metabolism tissues, controls body temperature and energy expenditure via thermogenesis through dissipating energy as heat [39]. BAT selectively expresses UCP1, which mediates thermogenesis by catalyzing the uncoupling of oxidative phosphorylation from ATP synthesis, in the inner membrane of the mitochondria [40]. WSE supplementation increased the expression of thermogenesis-related genes, including *UCP*1 and *PGC1α*, in BAT, when compared with the levels noted in the HFD-fed group. We also found that WSE supplementation stimulated the browning of scWAT by promoting UCP1 expression. The scWAT from the WSE-supplemented groups showed UCP1-positive multilocular adipocytes similar to the feature of BAT. When WAT browning is induced, white adipocytes transform to brown-like adipocytes that have high mitochondrial content. Browning of scWAT is considered a new therapeutic approach in treating obesity because it activates adaptive thermogenesis in response to cold exposure or dietary stimuli [9]. Our results indicate that WSE enhanced energy expenditure through increasing BAT activity and by stimulating scWAT differentiation into beige adipocytes. Activation of AMP-activated protein kinase (*AMPK*) and sirtuin 1 (*Sirt1*) result in activation of *PGC1α*, master regulator of mitochondrial biogenesis, and enhance mitochondrial oxidative function. This signaling pathway has been described as a regulating mechanism of inducing thermogenesis [41,42]. WSE could increase the expression of *PGC1α* and mitochondrial activity through activation of this signaling pathway, but the molecular mechanism of this effect needs further investigation. 

To investigate the mechanism whereby WSE enhances energy expenditure, we identified the major compounds present in WSE via LC-MS/MS analysis. The amounts of WFA and WNA found in 100 g of WSE were 366.5 mg and 203 mg, respectively. WFA, a major compound of WS, is known to improve nonalcoholic steatohepatitis [43] and exhibits antidiabetic effect by acting as a leptin-sensitizer [44]. We found that WFA increased UCP1 expression in the inner membrane of mitochondria and induced the differentiation of 3T3-L1 pre-adipocytes into beige adipocytes. WFA also significantly enhanced OCR in C2C12 cells, indicating that WFA improves mitochondrial respiration in myocytes. Whereas, WNA did not affect the differentiation of the 3T3-L1 pre-adipocytes into beige adipocytes (data not shown). Instead, it increased OCR in C2C12 cells and promoted myoblast differentiation by increasing of UCP2 protein expression in C2C12 cells. Whereas UCP1 plays important role in adaptive thermogenesis, UCP2, a UCP1 homologue that is expressed in various tissue including skeletal muscle, has primary function in the regulation of energy metabolism through reduction of mitochondrial ROS production [45,46]. Although the uncoupling proteins have distinct functions, they contribute to protecting metabolic syndrome [47]. Therefore, the increase in mitochondrial activity in the skeletal muscle of the WSE-supplemented groups was affected by the WFA and WNA contained in WSE. 

In conclusion, WSE ameliorated diet-induced obesity by enhancing energy expenditure via improving mitochondrial activity in skeletal muscle and adipose tissue. WFA and WNA, major compounds in WSE, were likely responsible for this anti-obesity effect. These data suggest that WS has potential as a new therapeutic agent for treating obesity. 

## Supplementary Materials

The following are available online at https://www.mdpi.com/2072-6643/12/2/431/s1, Figure S1: WSE reduces lipid accumulation in the liver and adipose tissue of HFD-fed mice; Figure S2: WSE improves insulin sensitivity in HFD-fed mice; Figure S3: WNA promotes the OCR in the C2C12 cells, Table S1: Primer sequence; Table S2: Diet formulation (g/kg).

## Abbreviations

WS, *Withania somnifera*; WSE, *Withania somnifera* 70% ethanol extract; HFD, high-fat diet; N, normal control diet; eWAT, epididymal adipose tissue; scWAT, subcutaneous adipose tissue; BAT, brown adipose tissue; OGTT, oral glucose tolerance test; IPITT, intraperitoneal insulin tolerance test; UCP1, uncoupling protein 1; UCP2, uncoupling protein 2; PGC1α, peroxisome proliferator-activated receptor gamma coactivator 1-alpha; Cidea, cell death activator CIDE-A; Dio2, type 2 deiodinase; Tfam, mitochondrial transcription factor A; VDAC, voltage-dependent anion channel; Nrf1, *nuclear respiratory factor 1; OCR, oxygen consumption rate; EE, energy expenditure; FCCP, carbonyl cyanide 4-(triflouromethoxy) phenylhydrazone;* WFA, withaferin A; WNA, withanolide A.
