# Supplementary material for: Withania somnifera Extract Enhances Energy Expenditure via Improving Mitochondrial Function in Adipose Tissue and Skeletal Muscle
Source: Nutrients. 2020 Feb 7;12(2):431. doi: 10.3390/nu12020431 (PMC7071232; doi:10.3390/nu12020431)

Supplementary Material

Supplementary Table S1. Primer sequence

Gene	Forward primer (5 ’→3 ’)	Reverse primer (5 ’→3 ’)
CD36	GATGACGTGGCAAAGAACAG	AAAGGAGGCTGCGTCTGTG
SCD-1	TTCTTGCGATACACTCTGGTGC	CGGGATTGAATGTTCTTGTCGT
SREBP1c	TGGATTGCACATTTGAAGACAT	GCCAGAGAAGCAGAAGAG
UCP1	GTGAACCCGACAACTTCCGAA	TGAAACTCCGGCTGAGAAGAT
PGC1α	TGGAGTGACATAGAGTGTGCTGC	CTC AAATATGTTCGCAGGCTCA
Dio2	AATTATGCCTCGGAGAAGACCG	GGCAGTTGCCTAGTGAAAGGT
Prdm16	CCACCAGCGAGGACTTCAC	GGAGGACTCTCGTAGCTCGAA
Cidea	TGCTCTTCTGTATCGCCCAGT	GCCGTGTTAAGGAATCTGCTG
UCP2	ATGGTTGGTTTCAAGGCCACA	TTGGCGGTATCCAGAGGGAA
Cox8b	TGTGGGGATCTCACCATAGT	GCAGGAGTACGATGAGTCCG
Tfam	GTCGCATCCCCTCGTCTATC	GCTGGAAAAACACTTCGGAATAC
Nrf1	AGCACGGAGTGACCCAAAC	TGTACGTGGCTACATGGACCT
C/EBPα	CAAGAACAGCAACGAGTACCG	GTCACTGGTCAACTCCAGCAC
PPARγ	TCGCTGATGCACTGCCTATG	GAGAGGTCCACAGAGCTGATT
FAS	GGAGGTGGTGATAGCCGGTAT	TGGGTAATCCATAGAGCCCAG
aP2	CCGCAGACGACAGGA	CTCATGCCCTTTCATAAACT
β-actin	GCAGGAGTACGATGAGTCCG	ACGCAGCTCAGTAACAGTCC
18S	CTCAACACGGGAAACCTCAC	CGCTCCACCAACTAAGAACG

Supplementary Table S2. Diet formulation (g/kg)

**Ingredient**	**N**	**HFD**	**HFD + 0.25% WSE**	**HFD + 0.5% WSE**
Casein	200	200	200	200
Corn oil	50	50	50	50
Lard	-	200	200	200
Cholesterol	-	5	5	5
Corn starch	350	145	145	145
Sucrose	300	300	300	300
Cellulose	50	50	50	50
Mineral	35	35	35	35
Vitamin	10	10	10	10
Methionine	3	3	3	3
Choline bitartate	2	2	2	2
WSE	-	-	2.5	5
Total	1,000	1,000	1,000	1,000

Supplementary figures

Supplementary Figure S1. WSE reduces lipid accumulation in the liver and adipose tissue of HFD-fed mice. (A) Food intake during the 10 weeks. (B) Organ weights. (C) H&E stained epididymal fat. (D) Measurement of adipocyte size. (E) H&E stained liver. (F) Hepatic total lpipid mesured by Folch method. (G) Expression of mRNA involved in the hepatic lipogenesis. Data represent mean ± SEM (n = 10). *p < 0.05; **p < 0.01; ***p < 0.001 compared to the HFD group.

Supplementary Figure S2. WSE improves insulin sensitivity in HFD-fed mice. (A) Blood glucose levels at the indicated time points after oral glucose administration. (B) Area under the curve of OGTT. (C) Blood glucose levels at the indicated time points after insulin injection. (D) Area under the curve of IPITT. Data represent mean ± SEM (n = 7). *p <0.05; **p < 0.01; ***p < 0.001 compared to the HFD group.

Supplementary Figure S3. WNA promotes the OCR in the C2C12 cells. (A) Oxygen consumption levels in WNA-treated C2C12 cells. (B) Quantification of basal respiration. (C) ATP production. (D) maximal respiration. (E) Spare capacity. (F) Protein expression of total MHC and UCP2 in C2C12 myogenic differentiated cells. Data represent mean ± SD (n = 5). *p <0.05, **p <0.01, ***p <0.001 compared to vehicle.

Supplementary Figure S1

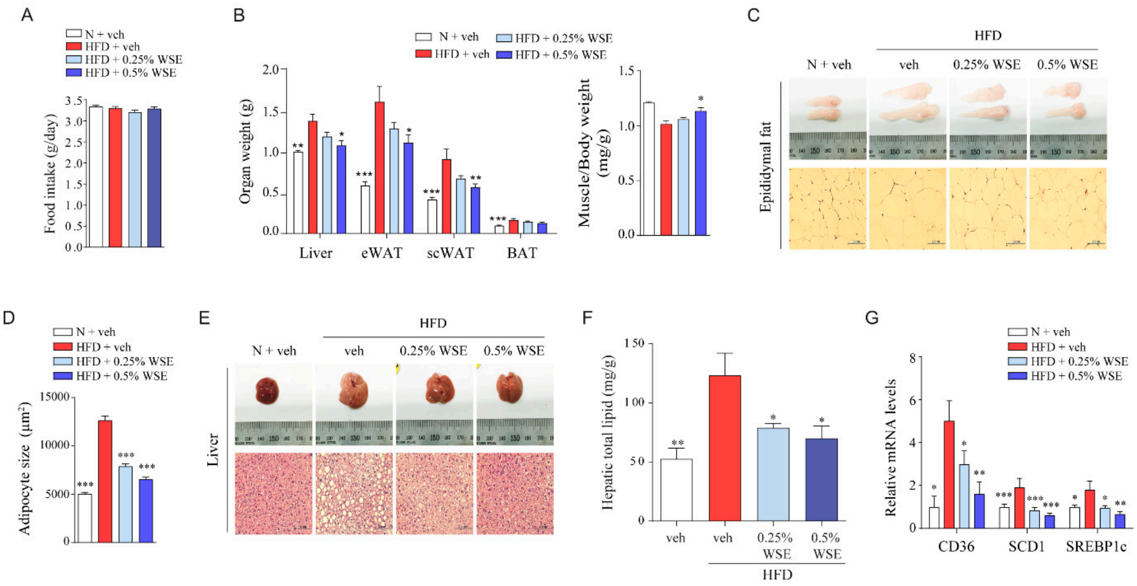

Supplementary Figure S2

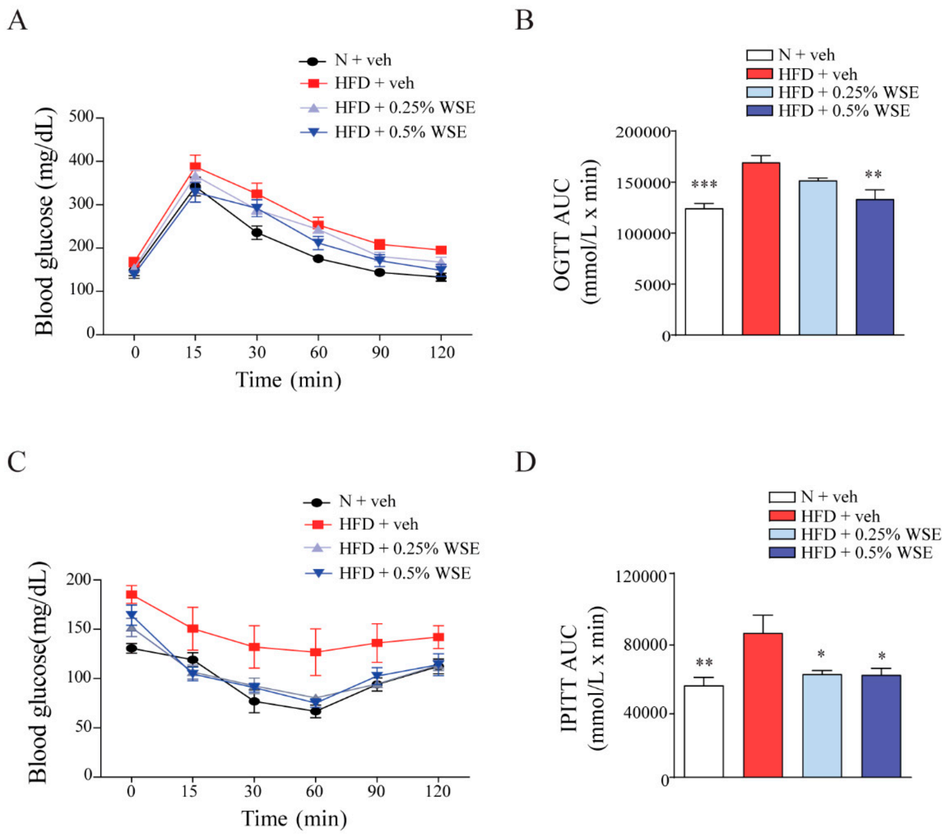

Supplementary Figure S3

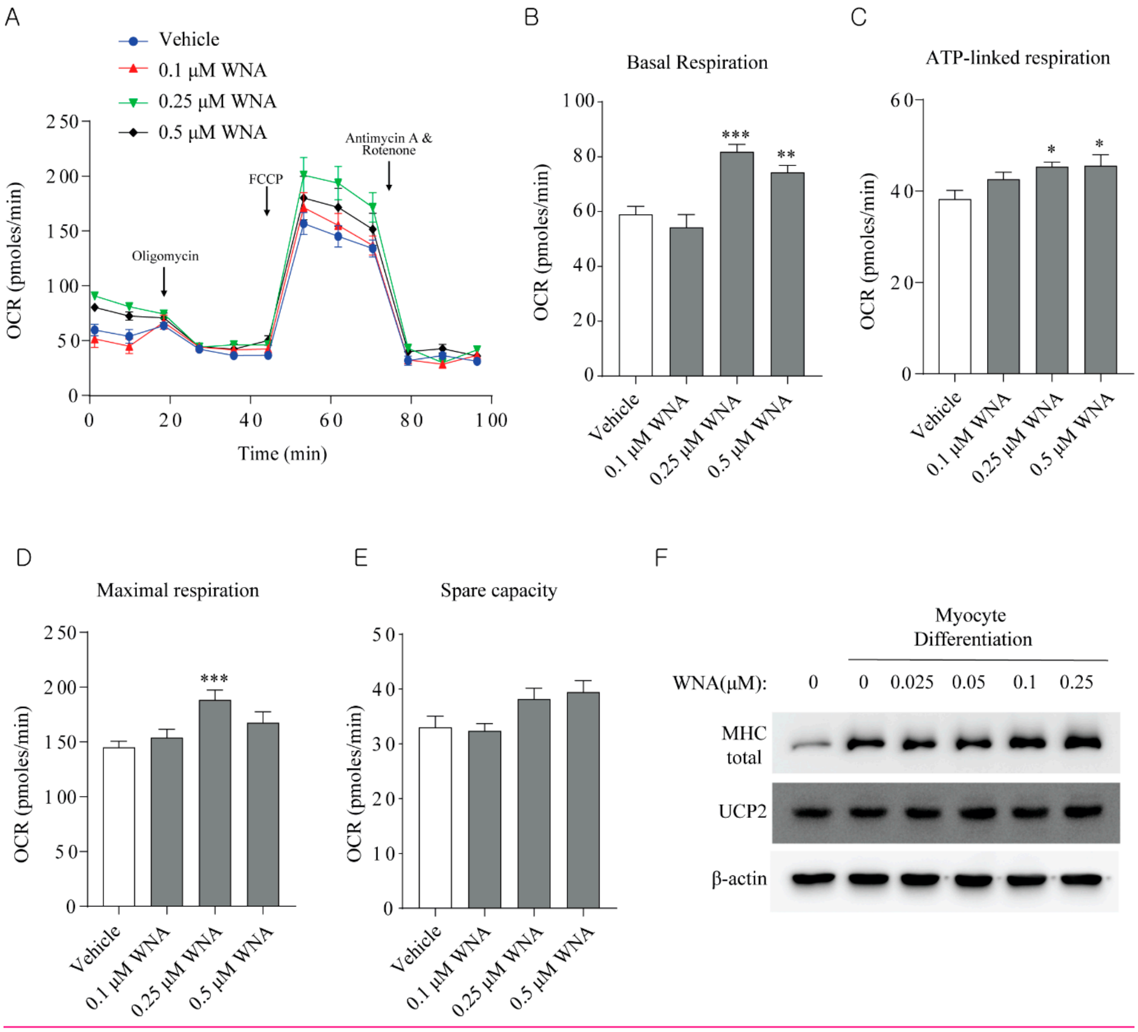

Supplement: Supplementary file 1 [file nutrients-12-00431-s001.pdf]
